# Supplementary material for: Metabolomic Plasma Profile of Chronic Obstructive Pulmonary Disease Patients
Source: Int J Mol Sci. 2025 May 9;26(10):4526. doi: 10.3390/ijms26104526 (PMC12111085; doi:10.3390/ijms26104526)
Supplement: Supplementary file 1 [file ijms-26-04526-s001.zip › ijms-3566470-supplementary.pdf]

SUPPLEMENTARY TABLES and FIGURES

Supplementary Table S1. Differential abundant metabolites between COPD patients and healthy subjects selected by t-test and corrected for False Discovery Rate (FDR) with a threshold of 0.1

	FC	log2(FC)	p.ajusted	-log10(p)	HMDB
4-Dodecylbenzenesulfonic acid	0,55159	-0,85833	1,46E-11	10,835	HMDB0059915
Urocanate	0,61582	-0,69941	4,09E-10	9,3888	HMDB0000301
2-Naphthalenesulfonic acid	0,49145	-1,0249	2,47E-09	8,6073	HMDB0255446
PPG n5	1,4777	0,5634	1,83E-07	6,7366	
Lignoceric acid (Tetracosanoic acid)	0,68266	-0,55077	2,52E-06	5,5995	HMDB0002003
2-Aminonicotinic acid	0,69599	-0,52287	2,95E-06	5,53	HMDB0061680
Nonadecanoic acid	0,73747	-0,43934	3,29E-06	5,4827	HMDB0000772
2-Hydroxyisocaproic acid &<br>2-Hydroxyhexanoic acid &<br>2-Ethyl-2-hydroxybutyric acid	1,4004	0,48579	3,69E-06	5,4334	HMDB0000665<br>HMDB0001624<br>HMDB0001975
Butyric acid	1,3465	0,42922	2,01E-05	4,696	HMDB0000039
Behenic acid	0,71115	-0,49178	2,76E-05	4,559	HMDB0000944
PPG n4	1,3394	0,42161	9,19E-05	4,0367	
Palmitate (Palmitic acid)	0,98824	-0,017063	0,00040182	3,396	HMDB0000220
Gluconic acid	1,3476	0,43038	0,00076913	3,114	HMDB0000625
DL-Glyceric acid	0,58371	-0,77667	0,0011543	2,9377	HMDB0000139
Tetraglyme	1,3506	0,43357	0,0027008	2,5685	HMDB0258894
1-Tetradecylamine	8,1374	3,0246	0,0027223	2,5651	HMDB0258887
CMPF	0,66779	-0,58253	0,0027223	2,5651	HMDB0061112

Bis(methylbenzylidene)sorbitol	1,1692	0,22555	0,0039794	2,4002	
Pentadecanoic acid	0,86574	-0,20799	0,0076701	2,1152	HMDB0000826
N-Acetylleucine	0,70363	-0,50712	0,0078515	2,105	HMDB0011756
Decanoylcarnitine	1,3	0,37847	0,0083332	2,0792	HMDB0000651
Lauroylcarnitine	1,3473	0,4301	0,0094106	2,0264	HMDB0002250
Pentanoate (valeric acid)	1,3473	0,43012	0,010171	1,9926	HMDB0000892
Octanoic acid (Caprylic acid)	1,4087	0,49439	0,011998	1,9209	HMDB0000482
DIPEA (Diisopropylethylamine)	0,28438	-1,8141	0,01576	1,8024	HMDB0247306
N-Methylglutamate	1,2104	0,27551	0,01695	1,7708	HMDB0062660
5-Valerolactone	1,1367	0,18486	0,01695	1,7708	HMDB0250981
Diethyl 2-Methyl-3-Oxosuccinate	0,74409	-0,42646	0,018576	1,731	HMDB0032306
Decanoic acid (capric acid)	1,2014	0,26474	0,024385	1,6129	HMDB0000511
Monoethyl malonate	1,3637	0,44755	0,028311	1,548	HMDB0000576
Octanoylcarnitine	1,2402	0,31061	0,028311	1,548	HMDB0000791
Diethanolamine	1,1448	0,19505	0,028311	1,548	HMDB0004437
N6-(Delta2Isopentenyl)Adenine	1,1802	0,23899	0,028623	1,5433	HMDB0245646
Hypotaurine	1,2653	0,33952	0,028701	1,5421	HMDB0000965
4-Coumarate	1,2218	0,28906	0,028701	1,5421	HMDB0002035
Bis(2-Ethylhexyl) Phthalate	0,75232	-0,41059	0,03591	1,4448	HMDB0249243
cis-7,10,13,16-Docosatetraenoic acid (adrenic acid)	1,3166	0,39682	0,03591	1,4448	HMDB0002226
Malate (Malic acid)	1,1859	0,24594	0,03591	1,4448	HMDB0000156
NP-013736	1,306	0,3852	0,040663	1,3908	
9-Decenoic acid (Caproleic acid)	1,1266	0,17197	0,044218	1,3544	HMDB0031003
14-Methylhexadecanoic acid	0,9101	-0,1359	0,044218	1,3544	HMDB0031067
beta-Hydroxyisovaleric acid	1,4108	0,49654	0,044967	1,3471	HMDB0000754
2-Hydroxytetradecanoic acid	0,83081	-0,26741	0,044967	1,3471	HMDB0002261

Methyl Vanillate	1,1797	0,23837	0,044967	1,3471	HMDB0240266
6-Methyl[1,2,4]triazolo[4,3-b]pyridazin-8-ol	1,1497	0,20121	0,044967	1,3471	
N-Acetylneuraminate	1,149	0,20039	0,045263	1,3443	HMDB0341204
1-Methyladenosine	1,1189	0,16204	0,045263	1,3443	HMDB0003331
Pseudouridine	1,1335	0,18073	0,046664	1,331	HMDB0000767
Eicosapentaenoate	0,70489	-0,50453	0,053609	1,2708	HMDB0001999
3-Hydroxybutanoate	1,2425	0,31326	0,053609	1,2708	HMDB0000357
Cotinine	1,6658	0,7362	0,06003	1,2216	HMDB0001046
Glutarate	1,1346	0,18221	0,062274	1,2057	HMDB0000661
Didemethylisoproturon (Isoproturon)	1,3084	0,38785	0,06284	1,2018	HMDB0253682
Palmitoylcarnitine	1,144	0,19406	0,063563	1,1968	HMDB0000222
Threitol	1,4509	0,53691	0,063632	1,1963	HMDB0004136
Biliverdin	1,4333	0,51937	0,063689	1,1959	HMDB0001008
DL-Dipalmitoylphosphatidylcholine (1,2 Dipalmitoylphosphatidylcholine)	1,109	0,14932	0,063689	1,1959	HMDB0255793
Hexanoylcarnitine	1,196	0,25822	0,071701	1,1445	HMDB0000756
Tyrosine	1,1805	0,23941	0,071701	1,1445	HMDB0000158
Methylthioadenosine	1,1486	0,19986	0,076897	1,1141	HMDB0001173
Maleate (Maleic acid)	0,84174	-0,24855	0,079472	1,0998	HMDB0000176
cis-5-Dodecenoic acid (5-dodecenoic acid)	1,1621	0,21677	0,079472	1,0998	HMDB0000529
Acetyl-L-carnitine (L-Acetylcarnitine)	1,1425	0,19226	0,079472	1,0998	HMDB0000201
cis-Aconitate	1,1349	0,18255	0,079472	1,0998	HMDB0000072
Arachidic acid (Icosanoic acid)	0,86114	-0,21568	0,079884	1,0975	HMDB0002212
Myristoylcarnitine	1,1483	0,19949	0,080033	1,0967	HMDB0254979
3-Hydroxymethylglutarate	1,1353	0,18305	0,080033	1,0967	HMDB0000355
DL-Histidine	2,8054	1,4882	0,080357	1,095	HMDB0250763

N-Acetylglutamate	1,1754	0,23315	0,081036	1,0913	HMDB0001138
Fumarate	1,1536	0,20613	0,08909	1,0502	HMDB0000134
3-Methylglutaconate	1,1381	0,18659	0,0902	1,0448	HMDB0000522
Xylitol	1,2353	0,30486	0,090328	1,0442	HMDB0002917
Dimethylarginine	1,11	0,15061	0,091574	1,0382	HMDB0251395
Glucose	1,1344	0,18194	0,095771	1,0188	HMDB0000122

Supplementary Table S2. Differential abundant metabolites between COPD patients and healthy subjects upon adjusting for age, sex, BMI and smoking status

	logFC	AveExpr	t	P.Value	adj.P.Val	B	HMDB
4-Dodecylbenzenesulfonic acid	-0,48498	1,87E-16	-8,0222	1,15E-13	3,73E-11	20,589	HMDB0059915
Urocanate	-0,45756	-5,05E-19	-7,2841	9,01E-12	1,46E-09	16,363	HMDB0000301
2-Naphthalenesulfonic acid	-0,47971	-3,47E-16	-7,1366	2,10E-11	2,27E-09	15,544	HMDB0255446
2-Hydroxyisocaproic acid<br>2-Hydroxyhexanoic acid<br>2-Ethyl-2-hydroxybutyric acid	0,33133	3,92E-16	5,7995	2,82E-08	2,29E-06	8,5956	HMDB0000665<br>HMDB0001624<br>HMDB0001975
Lignoceric acid	-0,352	-3,37E-16	-5,589	8,07E-08	4,56E-06	7,5885	HMDB0002003
2-Aminonicotinic acid	-0,32725	4,05E-16	-5,58	8,44E-08	4,56E-06	7,5457	HMDB0061680
PPG n5	0,345	1,81E-16	5,4972	1,27E-07	5,86E-06	7,1573	
Behenic acid	-0,33923	7,09E-17	-5,3055	3,18E-07	1,29E-05	6,2741	HMDB0000944
Nonadecanoic acid	-0,30634	4,34E-16	-5,236	4,43E-07	1,50E-05	5,9596	HMDB0000772
Butyric acid	0,30266	-2,31E-16	5,2259	4,64E-07	1,50E-05	5,9142	HMDB0000039
PPG n4	0,30217	1,73E-16	4,6707	5,75E-06	0,0001695	3,5178	
N-Acetylleucine	-0,297	-1,95E-16	-4,4536	1,46E-05	0,0003935	2,638	HMDB0011756
Tetraglyme	0,26912	1,74E-17	3,9125	0,0001282	0,0031946	0,5943	HMDB0258894
CMPF	-0,34425	-1,82E-16	-3,8307	0,0001748	0,0040454	0,3048	HMDB0061112
Diethyl 2-Methyl-3-Oxosuccinate	-0,254	5,73E-17	-3,7284	0,0002559	0,0052675	-0,050041	HMDB0032306
Gluconic acid	0,24119	2,88E-16	3,724	0,0002601	0,0052675	-0,065248	HMDB0000625
beta-Hydroxyisovaleric acid	0,23344	-3,38E-16	3,6647	0,0003232	0,00616	-0,26693	HMDB0000754
DL-Glyceric acid	-0,32747	-4,75E-17	-3,615	0,0003871	0,0069669	-0,43406	HMDB0000139
Maleate (Maleic acid)	-0,24591	-1,40E-16	-3,5761	0,000445	0,0075876	-0,56315	HMDB0000176
1-Tetradecylamine	0,47896	2,22E-16	3,5022	0,0005782	0,0091841	-0,80526	HMDB0258887
Azelate	-0,23277	-1,59E-16	-3,494	0,0005953	0,0091841	-0,83212	HMDB0000784

Pentadecanoic acid	-0,18134	2,61E-16	-3,4076	0,0008039	0,011839	-1,109	HMDB0000826
Pentanoate (Valeric acid)	0,23837	-4,11E-16	3,3425	0,0010045	0,01415	-1,3137	HMDB0000892
Octanoic acid (Caprylic acid)	0,25341	1,27E-16	3,3053	0,0011391	0,01527	-1,4291	HMDB0000482
Lauroylcarnitine	0,23406	4,16E-16	3,2953	0,0011782	0,01527	-1,4601	HMDB0002250
cis-7,10,13,16-Docosatetraenoic acid (Adrenic acid)	0,23967	-3,91E-16	3,2366	0,0014331	0,017596	-1,6393	HMDB0002226
4-Coumarate	0,19971	-2,02E-16	3,2286	0,0014713	0,017596	-1,6633	HMDB0002035
Decanoylcarnitine	0,25355	1,91E-16	3,2187	0,0015207	0,017596	-1,6935	HMDB0000651
N6-(Delta2Isopentenyl)Adenine	0,1891	-7,77E-17	3,1499	0,0019042	0,021275	-1,8986	HMDB0245646
Bis(methylbenzylidene)sorbitol	0,17087	-3,80E-17	3,0859	0,0023408	0,02528	-2,0864	
2-Hydroxytetradecanoic acid	-0,20222	3,26E-16	-3,0292	0,0028024	0,029289	-2,2496	HMDB0002261
Diethanolamine	0,16912	-2,52E-16	2,9657	0,0034181	0,034608	-2,4292	HMDB0004437
Triethanolamine	-0,20333	-2,22E-16	-2,9446	0,0036484	0,035821	-2,4881	HMDB0032538
Palmitate (Palmitic acid)	-0,057507	1,20E-17	-2,8987	0,0041996	0,04002	-2,6148	HMDB0000220
3-Hydroxybutanoate	0,19328	-3,45E-16	2,8806	0,004437	0,041074	-2,6643	HMDB0000357
Methyl Vanillate	0,17421	1,76E-16	2,8574	0,0047594	0,042835	-2,7273	HMDB0240266
Palmitoylcarnitine	0,15777	-9,61E-17	2,7673	0,0062251	0,054512	-2,9676	HMDB0000222
Octanoylcarnitine	0,21997	-3,97E-16	2,7358	0,0068289	0,057289	-3,0502	HMDB0000791
Biliverdin	0,36691	-5,28E-17	2,7324	0,0068959	0,057289	-3,0589	HMDB0001008
DIPEA (Diisopropylethylamine)	-0,25027	-1,49E-16	-2,6565	0,0085854	0,069318	-3,2537	HMDB0247306
9-Decenoic acid (Caproleic acid)	0,17782	-1,38E-16	2,6445	0,0088837	0,069318	-3,284	HMDB0031003
Arachidic acid (Icosanoic acid)	-0,13178	5,43E-16	-2,6405	0,0089857	0,069318	-3,2941	HMDB0002212
cis-5-Dodecenoic acid (5-dodecenoic acid)	0,17653	5,36E-17	2,6283	0,0093019	0,070089	-3,3248	HMDB0000529
Bis(2-Ethylhexyl) Phthalate	-0,22346	-3,18E-17	-2,6118	0,0097443	0,071754	-3,3659	HMDB0249243
Malate (Malic acid)	0,14906	-4,94E-17	2,5595	0,011279	0,079672	-3,495	HMDB0000156
Acetyl-L-carnitine (L-Acetylcarnitine)	0,15737	-1,02E-16	2,5585	0,011311	0,079672	-3,4975	HMDB0000201

14-Methylhexadecanoic acid	-0,12759	-2,36E-16	-2,5446	0,011757	0,080064	-3,5315	HMDB0031067
4-Hydroxybenzaldehyde	0,16986	1,57E-19	2,5414	0,011861	0,080064	-3,5393	HMDB0011718
NP-013736	0,18365	-3,33E-16	2,5317	0,012182	0,080547	-3,5627	
3-Hydroxybenzaldehyde	0,16811	1,09E-16	2,515	0,012753	0,082034	-3,6029	
Monoethyl malonate	0,19218	2,82E-17	2,5105	0,012913	0,082034	-3,6139	HMDB0000576
N-Methylglutamate	0,14171	2,34E-16	2,4851	0,013838	0,086224	-3,6746	HMDB0062660
Sara Unknown 2 (nv)	0,15133	-4,43E-17	2,4699	0,014421	0,087858	-3,7107	
3-Methylglutaconate	0,15407	-4,58E-16	2,4642	0,014643	0,087858	-3,724	HMDB0000522
Decanoic acid (capric acid)	0,15908	-2,78E-16	2,4294	0,016078	0,094715	-3,8057	HMDB0000511
Tyrosine	0,14632	2,34E-16	2,4102	0,016922	0,097908	-3,8503	HMDB0000158

Supplementary Table S3. Pathway analysis of differential abundant metabolites between COPD patients and healthy subjects (upon adjusting for age, sex, BMI and smoking status) based on KEGG and SMPDB libraries

KEGG library

Pathway Name	Match Status	p	-log(p)	Holm p	FDR	Impact
Fatty acid biosynthesis	3/47	0,0057767	2,2383	0,46214	0,24873	0,01473
Butanoate metabolism	2/15	0,0062182	2,2063	0,49124	0,24873	0
Pentose phosphate pathway	2/23	0,014434	1,8406	1	0,38491	0,04419
Glyoxylate and dicarboxylate metabolism	2/32	0,027136	1,5665	1	0,44847	0,07937
Phenylalanine, tyrosine and tryptophan biosynthesis	1/4	0,03264	1,4862	1	0,44847	0,5
Biosynthesis of unsaturated fatty acids	2/36	0,033832	1,4707	1	0,44847	0
Fatty acid degradation	2/39	0,039241	1,4063	1	0,44847	0
Phenylalanine metabolism	1/8	0,064294	1,1918	1	0,64294	0
Glycerolipid metabolism	1/16	0,12475	0,90395	1	0,99803	0,09346
Histidine metabolism	1/16	0,12475	0,90395	1	0,99803	0,12295
Ubiquinone and other terpenoid-quinone biosynthesis	1/18	0,13929	0,85607	1	1	0
Citrate cycle (TCA cycle)	1/20	0,15361	0,81358	1	1	0,04412
Pyruvate metabolism	1/23	0,17468	0,75777	1	1	0,0283
Porphyrin metabolism	1/31	0,2285	0,64111	1	1	0,07453
Glycine, serine and threonine metabolism	1/33	0,24145	0,61718	1	1	0,02475
Fatty acid elongation	1/39	0,27908	0,55428	1	1	0

Tyrosine metabolism	1/42	0,29724	0,5269	1	1	0,13972

SMPDB library

Pathway Name	Match Status	p	-log(p)	Holm p	FDR	Impact
Fatty Acid Biosynthesis	4/33	5,52E-04	3,2577	0,054687	0,054687	0,12432
Beta Oxidation of Very Long Chain Fatty Acids	2/13	0,01104	1,957	1	0,5465	0
Malate-Aspartate Shuttle	1/7	0,087025	1,0604	1	1	0,28571
Fatty Acid Metabolism	2/40	0,090726	1,0423	1	1	0
Ketone Body Metabolism	1/12	0,14485	0,83909	1	1	0
Catecholamine Biosynthesis	1/14	0,16701	0,77725	1	1	0
Butyrate Metabolism	1/16	0,18865	0,72435	1	1	0
Alpha Linolenic Acid and Linoleic Acid Metabolism	1/16	0,18865	0,72435	1	1	0,11111
Transfer of Acetyl Groups into Mitochondria	1/18	0,20976	0,67827	1	1	0,30905
Oxidation of Branched Chain Fatty Acids	1/22	0,25048	0,60123	1	1	0
Glycerolipid Metabolism	1/23	0,26035	0,58444	1	1	0
Phenylalanine and Tyrosine Metabolism	1/25	0,27974	0,55325	1	1	0,22124
Citric Acid Cycle	1/26	0,28925	0,53872	1	1	0
Gluconeogenesis	1/30	0,32617	0,48656	1	1	0,017794
Fatty Acid Elongation In Mitochondria	1/33	0,35268	0,45262	1	1	0
Porphyrin Metabolism	1/36	0,37823	0,42224	1	1	0,054945
Pyruvate Metabolism	1/37	0,38654	0,41281	1	1	0

Warburg Effect	1/49	0,47855	0,32007	1	1	0
Glycine and Serine Metabolism	1/50	0,48561	0,31371	1	1	0
Tyrosine Metabolism	1/55	0,51961	0,28432	1	1	0
Bile Acid Biosynthesis	1/59	0,54531	0,26336	1	1	0

Supplementary Figure S1. Heatmap of the 56 significant differentially abundant metabolites (DAMs) distinguishing COPD patients from control subjects

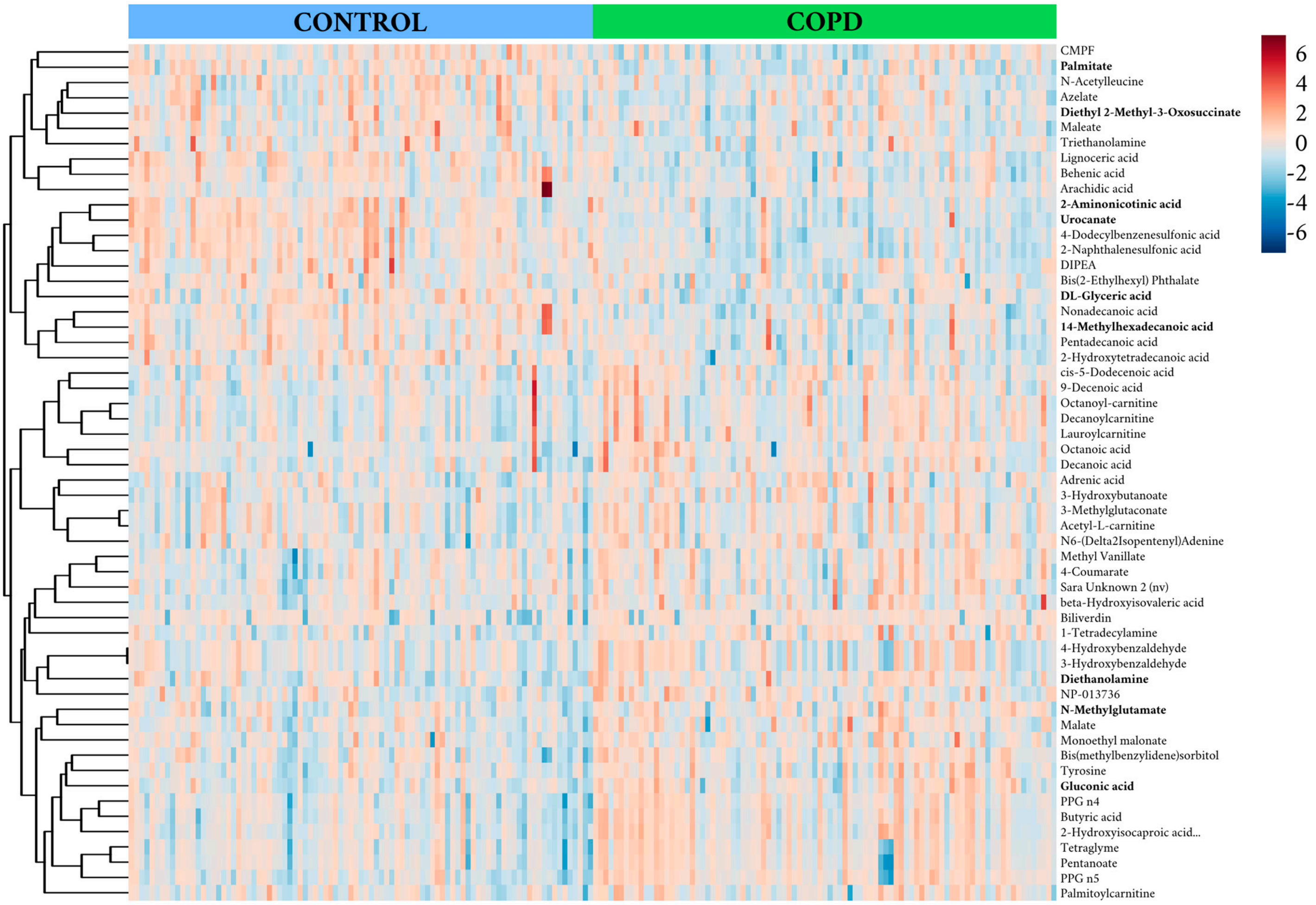

This heatmap was generated using MetaboAnalyst 6.0, employing Euclidean distance as the similarity metric and complete algorithm for hierarchical clustering. Differentially abundant metabolites (DAMs) are listed on the left; the 10 highlighted in bold represent the final endogen (without xenobiotics) signature selected in further analysis.

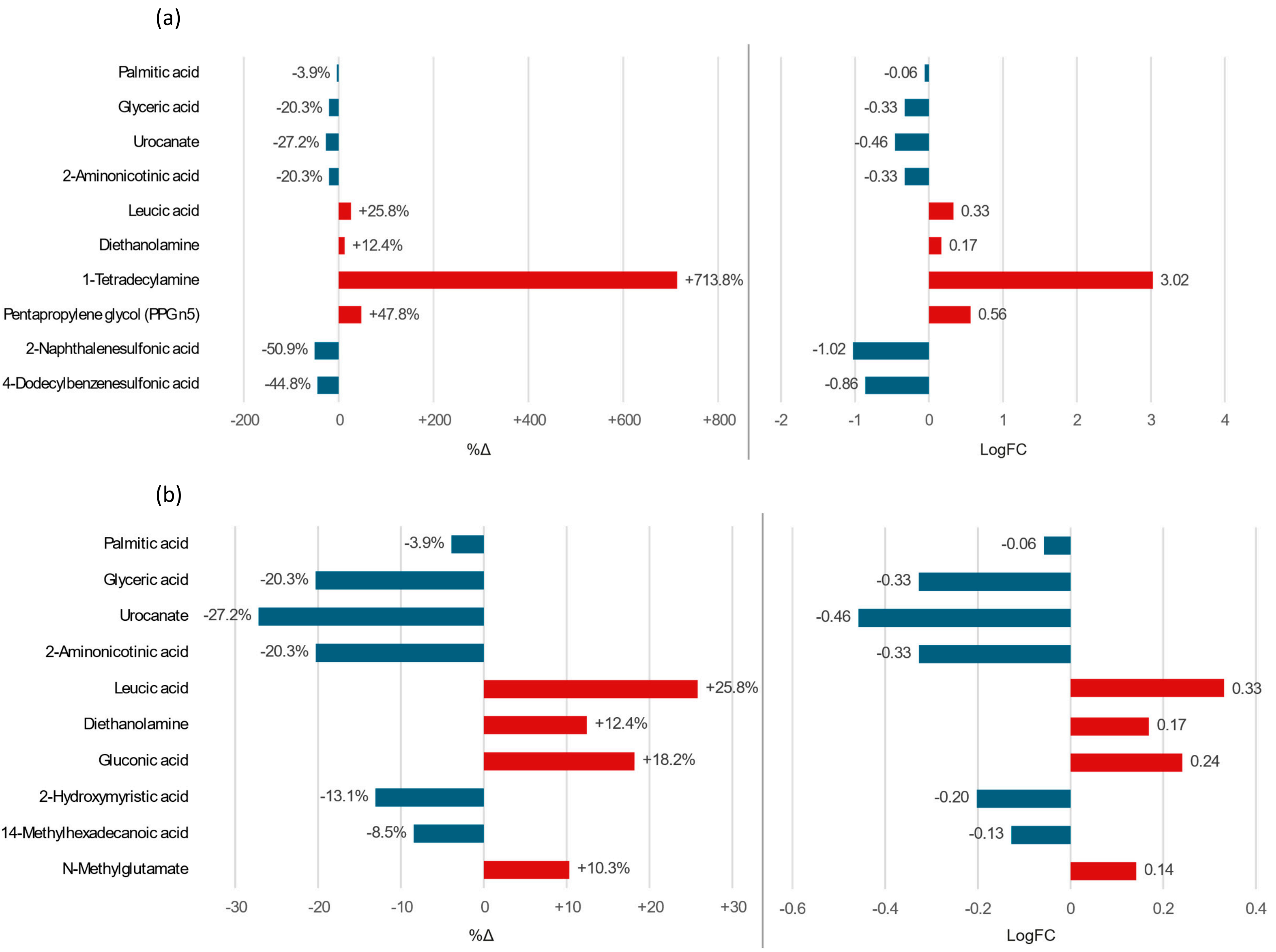

Supplementary Figure S2. Bar graph of the selected metabolites for the metabolomic panel to discriminate COPD patients from control subjects (a) with xenobiotics included; and (b) excluding xenobiotics. Abbreviations: %Δ, percent change; FC, Fold change.
